# The predominant learning approaches of medical students

**DOI:** 10.1186/s12909-018-1122-5

**Published:** 2018-01-18

**Authors:** Sonali Prashant Chonkar, Tam Cam Ha, Sarah Shan Hang Chu, Ada Xinhui Ng, Melissa Li Shan Lim, Tat Xin Ee, Mor Jack Ng, Kok Hian Tan

**Affiliations:** 1 KK Women’s and Children’s Hospital, Singapore, Singapore; 2 Duke-NUS Medical School, Singapore, Singapore; 3 Yong Loo Lin School of Medicine, National University of Singapore, Singapore, Singapore

**Keywords:** Learning approaches, Medical students, Predominant learning styles

## Abstract

**Background:**

By identifying medical students’ learning approaches and the factors that influence students’ learning approaches, medical schools and health care institutions are better equipped to intervene and optimize their learning experience. The aims of our study is to determine the predominant learning approach amongst medical students on a clinical posting in a hospital in Singapore and to examine the demographic factors that affect their learning approach.

**Methods:**

The Approaches and Study Skills Inventory for Students (ASSIST) questionnaire was administered to 250 medical students from various medical schools on clinical attachment to the Obstetrics and Gynaecology (O&G) department of KK Women’s and Children’s Hospital (KKH) Singapore between March 2013 and May 2015 to determine students’ predominant learning approaches. Multinomial logistic regression was used to examine the association between demographic factors (age, gender and highest education qualification) and predominant learning approach. A cut-off of *p* < 0.05 was used for statistical significance.

**Results:**

Amongst 238 students with one predominant learning approach, 96 (40.3%) and 121 students (50.8%) adopted the deep and strategic approach respectively, whilst only 21 (8.8%) adopted the surface approach. Male students appeared less likely to adopt the strategic learning approach than female students (*p* value = 0.06). Predominant learning approaches were not influenced by demographic characteristics such as age, gender and highest educational qualifications.

**Conclusions:**

This study provided insight into the learning approaches of a heterogeneous group of medical students in Singapore. While it is encouraging that the majority of students predominantly utilised the deep and strategic learning approach, there was a significant proportion of students who utilised the surface approach. Interventions can be explored to promote deeper learning amongst these students.

## Background

A learning approach is the strategy that one adopts while seeking knowledge. According to Entwistle et al., the way a student approaches a learning situation is not inherent, but an acquired trait or strategy dependent on the learning context or situational demands [1]. Approaches to learning might change over the several years of a medical degree programme if suitable efforts were made to promote that change. As such, a holistic understanding of the predominant learning approaches and how the various demographic factors may possibly influence the learning approaches of medical students is crucial in helping educators to intervene and create a more favourable learning environment to enhance student learning and better prepare them for the future.

Three learning approaches have been identified, namely the deep, strategic and surface apathetic approach. Deep approach emphasizes understanding concepts, relating and having an interest in ideas. Surface apathetic approach, on the other hand, is a syllabus bound superficial method of learning, where emphasis is on rote memorisation, with a lack of understanding and intention to only cope minimally with the course. With the strategic approach, students are motivated to achieve the highest scores possible. This involves good time management and study organisation, however, this type of learning may result in fragmented understanding of contents, with poorer integration across topics as compared to the deep approach [2].

As medical school curriculum involves critical analysis of new ideas, linking of new ideas to already known concepts and principles, and using this knowledge for problem solving in unfamiliar contexts, it is favourable for deep and strategic learning approaches to be the predominant learning approach adopted by medical students [3]. In contrast, the surface apathetic approach is less favourable as medical students will less effectively retain large amounts of medical content. Previous studies have established a generally positive correlation between the deep learning approach and quality of student learning and the opposite for the surface apathetic approach [4].

Several studies [3, 5–12] have been conducted on various populations of medical school students globally, examining students’ approaches to learning. These include three Sri Lankan studies conducted with study populations of Year 2 pre-clinical medical students [3] and mixtures of pre-clinical, clinical and post-graduate medical students [5, 6]. There were also studies of undergraduate MD pre-clinical students from Xavier Medical School of Medicine in Aruba in the Caribbean [7]; students across the five-year medical education programme study evaluating the success of a redesigned medical education curriculum in encouraging a deep and deterring a surface approach to learning in UK [8]; medical undergraduate students and pre-clinical medical students in Nepal [9, 10]; incoming students into the Medicine Faculty at the University of Alberta, Canada [11] and a mix of year 1, 2 and 5 medical students in the University of Gadjah Mada, Indonesia [12].

KK Women’s and Children’s Hospital (KKH), formerly known as “Kandang Kerbau Hospital”, is the largest restructured hospital specialising in healthcare for women and children in Singapore. It is the main institute where medical students undergo their clinical attachment in Obstetrics and Gynaecology (O&G). The hospital has students from two local universities National University of Singapore Yong Loo Lin School of Medicine (YLL), Duke-NUS Medical School (Duke-NUS), as well as foreign universities. YLL is Singapore’s oldest medical school with British origins and offers an undergraduate five-year medical education programme to students, with 2 years of pre-clinical education and clinical rotations starting from third year onwards. Duke-NUS, a USA-style medical school is Singapore’s only graduate medical school, and offers a four-year programme for post-graduates, with clinical rotations beginning in the second year. The majority of foreign elective students came from European universities, such as University of Liverpool, Royal College of Surgeons in Ireland, Queen’s University Belfast, University College Dublin, First Moscow State, Imperial College London, King’s College London, Trinity College Dublin, Karolinska Institute, Cambridge University, University of Sheffield, University College Cork, Edinburgh University; and also Australian Universities, such as University of Western Australia, University of Adelaide, The University of Sydney and University of Melbourne. The students from YLL and Duke-NUS are both doing the O&G curriculum as their core, while the students from foreign universities are on elective placement. Among YLL students, 100% of them are ages 25 and below and have obtained GCE ‘A’ Levels as their highest educational qualification. Among the Duke-NUS students, 100% of them are aged above 25 and their highest educational qualification is above ‘A’ levels. Among the foreign elective students, 11.4% are aged 25 and above, and 36.4% have above ‘A’ levels as their highest educational qualification.

Compared to previous studies in which study population relates to a single or predominant medical school [3, 5–12], the medical student population in our hospital is heterogeneous with diverse medical school background. This merits a study within our hospital in understanding learning approaches of medical students. The demographics and backgrounds of medical students among the 2 local medical schools in Singapore are also diverse in terms of nationality, backgrounds and previous educational qualifications [13] which may further influence their choice of learning approach. In addition, medical education in Singapore is not standardized among the various medical schools in terms of curriculum, assessment and teaching methods, meaning that the student population may have experienced a range of medical curriculum prior to taking part in the study and could be from schools with very different medical educational approaches. Among the three cohorts, the third cohort consists of foreign students on their elective placements. They might have been exposed to very different medical educational approaches. Though they comprise only 18.5% of the total study population, their previous educational experiences and cultural backgrounds could potentially have influenced their learning styles.

The aims of this study was conducted to determine the predominant type of learning approach amongst the heterogeneous mix of medical students in Singapore and the demographic factors which may affect their learning approach. We aim to investigate if the predominant learning approach that a medical student undertakes is influenced by demographic factors such as age, gender and highest educational qualifications. Through identifying students’ predominant learning approach, we hope to provide insights that will help educators to tailor their curriculum to empower students to shift towards the deep learning approach.

Our study serves to establish a baseline to identify whether this background contributes to their predominant learning approach at the point of entry into the O&G placement. As the learning objectives for the O&G placement are the same regardless of whether a student enters as an elective or as part of core curriculum, we believe that there are more appropriate learning approaches for these learning objective. Therefore, if we are able to identify the learning approaches, we can then better tailor interventions to modify and improve the learning approach to a more apt one if need be.

## Methods

### Participants

The study population comprised 250 medical students from various medical schools who attended O&G rotation at KKH between March 2013 and May 2015.

### Questionnaire

We used the Approaches and Study Skills Inventory for Students (ASSIST) questionnaire as a tool for evaluating the learning approaches of students. The ASSIST questionnaire [14], developed by Entwistle and McCune, is a revised version of the Approaches to Studying Inventory (ASI). The ASSIST questionnaire has been validated in various samples and cultures globally, including amongst eastern cultural population such as Chinese university students and also across the western student population in Britain and Scotland. [15] ASSIST is an appropriate instrument and the results are valid as indicated by the scores obtained in the main scales and subscales [16]. The Approaches and Study Skills Inventory for Students (ASSIST) questionnaire was used to investigate the students’ preferred learning approaches [17] given the diverse backgrounds of our student population sample in Singapore.

Students completed the ASSIST questionnaire within the first 2 weeks of their rotation. It comprises 52 (16 + 16 + 20) questions, each scored on a Likert scale of 1(low)- 5(high), with 16 questions pertaining to surface and deep learning each and 20 questions relating to strategic learning. An example of a question assessing deep approach is “I try to relate ideas I come across to those in other topics or other courses whenever possible.”, strategic approach is “I think I’m quite systematic and organised when it comes to revising for exams.”, and surface approach is “I find I have to concentrate on just memorising a good deal of what I have to learn.”

The scores for sets of 4 were combined into 13 subscales and further grouped to give each respondent a score each for deep, strategic and surface approach. Although the scoring procedure in the ASSIST questionnaires states that scores on the three main approaches are created by adding together the subscale scores which contribute to each approach, we found that the number of subscales vary among the learning approaches. There are four subscales in both the deep and surface approaches and five subscales in the strategic approach. As a result, we have decided to compute the mean after adding the subscale scores of each learning approach and take the highest mean to be indicative of the student’s predominant learning approach.

Several studies [6, 18, 19] in the literature that have used the ASSIST questionnaire have also used mean scores to determine the predominant learning approach used by students. For instance, Byrne et al. from Dublin City University, Dublin, Ireland in 2001 studied the relationship between learning approaches and learning outcomes in Irish accounting students and used the ASSIST questionnaire to measure students’ approaches to learning by computing the mean scores of each learning approach [18]. A study from Turkey conducted by Cebeci et al. [19] used mean SD and Mann Whitney u test to analyse their results. Another study by Samara Koon et al. [6] used two questionnaires to validate their study with the ASSIST questionnaire using highest mean score to determine predominant learning approach. We therefore also used highest mean score to determine the student’s predominant learning approach.

There were 12 students who were found to have more than one dominant approach; these students were excluded from the analysis, leaving a sample of 238 students. Out of the 12 students, 4 had all 3 predominant approaches, 4 have deep and surface, 3 deep and strategic, and 1 strategic and surface. This division makes it very difficult to analyse the data in any appreciative way, more so because the number is less than 5% of the total study population. Hence, we excluded these 12 from our analysis. We examined the association between age, gender and higher educational qualification with predominant learning approaches. Age was further explored by examining two groups; 25 years old and below and above 25 years old.

### Ethical considerations

The study was ethically approved and given the exempt status by our institution’s Centralised Institutional Review Board of SingHealth (CIRB) committee. All students participated voluntarily and a declaration of informed consent was obtained before participating in the study. The CIRB reference number for our study is 2013/232/D.

### Statistical analysis

We removed from the analyses students who had multiple learning approaches (*n* = 12), leaving us with 238 students. Out of the 12 students, 4 had all 3 predominant approaches, 4 have deep and surface, 3 deep and strategic, and 1 strategic and surface. This division makes it very difficult to analyse the data in any appreciative way, more so because the number is less than 5% of the total study population.

Descriptive statistics were used to describe participants’ demographic variables. Categorical variables were reported as proportions and percentages. Multinomial logistic regression was used to examine the association between the demographic factors (age, gender, and highest education qualification) and predominant learning approach, allowing the modelling of the strategic and deep approaches (reference group = surface). For the analysis, a cut- off of *p* < 0.05 was used to determine statistical significance. Data was analysed using SPSS (version 23.0 for Windows, Armonk, NY: IBM Corp).

## Results

### Demographic data

The demographics of the study participants are shown in Table 1. The mean age of participants was 23.9 years. Of the 238 participants, 179 (75.2%) were aged 25 years and below and 59 (24.8%) above 25 years. 96 of 238 participants were male (40.3%) and 142 (59.7%) were female. 135 of 238 (56.7%) participants had General Certificate of Education (GCE) ‘A’ levels as their highest educational qualification upon entering medical school and 103 (43.3%) had above ‘A’ Levels as their highest qualification.

**Table 1:** Student demographics (*n* = 238)

Variable		n	%
Age (years)	≤ 25	179	75.2
	> 25	59	24.8
Gender	Male	96	40.3
	Female	142	59.7
Current Medical School	YLL	107	45.0
	Duke-NUS	87	36.6
	Foreign Universities	44	18.5
Highest Educational Qualification	General Certificate of Education (GCE) ‘A’ Levels	135	56.7
	Above ‘A’ Levels	103	43.3

### Learning approaches

Paired *t*-tests were conducted to determine whether there were significant differences between the mean scores within each learning approach. There was a significant difference between deep (M = 0.40, SD = 0.49) and surface (M = 0.09, SD = 0.28) approaches; *t*(237) = 7.75, *p* < 0.001) and between strategic (M = 0.51, SD = 0.50) and surface (M = 0.09, SD = 0.28) approaches; *t*(237) = 9.98, *p* < 0.001). These results suggested that there is a statistically significant difference between the mean scores of deep versus surface, and strategic versus surface.. However, there is no statistical difference between deep (M = 0.40, SD = 0.49) and strategic (M = 0.51, SD = 0.50) approaches; *t*(237) = − 1.70, *p* = 0.09. Amongst the 238 students, 96 (40.3%) adopted the deep and 121 (50.8%) adopted the strategic as their predominant learning approach, while 21 (8.8%) of students adopted the surface learning approach. Male students are deemed to be less likely than female students to exhibit strategic learning, (*p* = 0.06). The highest education qualification attained (A Levels versus above A Levels) was not significantly associated with the predominant learning approach (*p* = 0.50). There was no significant difference between the predominant learning approach of respondents above 25 years old and those below 25 years (*p* = 0.40).

Tables 2, 3 & 4 show the results of the multinomial regression analysis examining correlates of predominant learning approach. There were no demographic variables that were significantly associated with predominant learning approaches. Male students appeared less likely to adopt the strategic learning approach than female students (*p* = 0.06), although this effect was not statistically significant. Even when the objectives of learning was divided into core and elective, there was no significant difference in the learning approaches (YLL and Duke-NUS vs. foreign universities).

**Table 2:** Student demographics characterised by school (*n* = 238)

Current medical school	Age		Gender		Highest educational qualification	
	≤ 25	> 25	Male	Female	GCE ‘A’ Levels	Above ‘A’ Levels
	n (%)	n (%)	n (%)	n (%)	n (%)	n (%)
YLL (*n* = 107)	107 (100)	0 (0)	47 (43.9)	60 (56.1)	107 (100)	0 (0)
Duke-NUS (*n* = 87)	33 (37.9)	54 (62.1)	43 (49.4)	44 (50.6)	0 (0)	87 (100)
Foreign Universities (*n* = 44)	39 (88.6)	5 (11.4)	6 (13.6)	38 (86.4)	28 (63.6)	16 (36.4)

*GCE* general certificate of education

**Table 3:** Multinomial regression model: predicting likelihood of predominant learning approach (*n* = 238)

	Characteristic	Odds ratio	95% CI	*P* value
Strategic	Age 25 and below	0.52	0.11–2.39	0.40
	Male	0.40	0.16–1.05	0.06
	General Certificate of Education A-Levels	1.52	0.44–5.23	0.50
Deep	Age 25 and below	0.87	0.20–3.91	0.86
	Male	1.29	0.49–3.39	0.60
	General Certificate of Education A-Levels	0.58	0.17–1.97	0.38

**Table 4:** Association between age, gender, highest educational qualifications and predominant learning approach as described by odds ratios (ORs) for multinomial logistic regression model

	Predominant learning approach			
	Strategic		Deep	
Characteristic	OR (95% Cl)	*p* value	OR (95% Cl)	*p* value
Age				
25 years and below	0.52 (0.11–2.39)	0.40	0.87 (0.20–3.91)	0.86
Above 25 years	Ref		Ref	
Gender				
Male	0.40 (0.16–1.05)	0.06	1.29 (0.49–3.39)	0.60
Female	Ref		Ref	
Highest Education Qualification				
General Certificate of Education A-Levels	1.52 (0.44–5.23)	0.50	0.58 (0.17–1.97)	0.38
Above A-Levels	Ref		Ref	

Ref: reference group

## Discussion

Our study sampled local and foreign students from local and overseas medical schools undergoing a specific clinical rotation in a public hospital. This is the first-of-its-kind medical education study applied to a heterogeneous population from different medical schools within the same hospital setting. This distinguishes our study from its predecessors where students sampled were from the same medical school and studies were conducted internally within universities or hospitals affiliated to universities to examine the learning approaches of their own students.

Our study found that the majority of students used deep and strategic learning approaches, with the predominant approach to learning being the strategic approach. In a study conducted by Shankar et al. [7] on undergraduate medical students in a medical school in Aruba, the majority used deep and strategic approaches to learning, with the median scores for deep, strategic and surface approach being 60, 73 and 52 respectively. The study population consisted of students mainly from the United States and Canada admitted to the undergraduate medical (MD) program. Another study conducted by Samarakoon et al. [6] in Sri Lanka also found that the strategic learning was the predominant learning approach in all three groups of pre-clinical, clinical and postgraduate students. In this study, the learning styles and approaches to learning in cohorts of undergraduate students in first (preclinical) year and final (clinical) year in the University of Colombo as well as postgraduate trainees of the Postgraduate Institute of Medicine, University of Colombo, Sri Lanka, were analysed. Both these studies similarly found the preference for deep and strategic learning amongst the students and also the predominance of strategic learners, which concurs with our study on medical students in our hospital. The predominant deep and strategic learning approaches amongst these student populations may be due to the global trend towards encouraging deeper learning from medical students through a shift in medical education paradigm from didactic lectures to integrated problem-based learning [20]. Problem-based learning is a student-centered activity known to promote deep learning, unlike conventional teaching methods [8, 10]. In addition, the predominant deep and strategic learning approach may also relate to the inherent internal motivation and interest of medical students in the medical field [21].

Despite the differences in sample populations in other studies [6, 9] with ours, all three-study populations consistently showed an overall preference for the strategic approach to learning. This suggests that despite individual schools’ efforts to promote deep learning, majority of students studying medicine irrespective of culture or school, appear to prefer assessment-oriented strategic learning rather than deep learning. We posit that it could be potentially due to the need to cope with the heavy content, higher workload and tight course schedules in medical education that would require one to have good time management and organisation skills.

However, in Shah et al.’s [9] study of the learning approach among health sciences students which included medical students in a medical college in Nepal, he found that majority of the medical students adopted the deep learning as their predominant learning approach. A possible explanation for this difference may be that the students’ learning approaches have already been established prior to university entry [9]. Learning approaches of medical students could have already been shaped by the quality of their teaching-learning environment and assessment procedures [18] in their pre-university years. As pre-university education in Singapore is largely didactic and lecture based, pre-university students are encouraged to use strategic learning. Many pre-university students in Singapore also attend supplementary private tuition classes [22]. Attending private tuition classes reinforces the strategic and surface learning approach of students, as private tutors primarily promote exam-oriented learning to yield better student performance statistics and increase their own tutor credibility [14]. Thus, Singapore’s pre-university education may be more competitive then Nepal’s as greater emphasis is placed on achieving good grades, a pre-requisite to entering medical school. In addition, the predominant deep learning approach in medical students may also be a reflection of the nature of medical school education in Nepal. Hence, it would be beneficial to compare how the local medical curricular differ from others to better modify our curriculum to promote deep learning.

Our study results showed that difference in age was not significantly related to the predominant learning approaches adopted by the students. This aligns with the findings from the study of pre-clinical undergraduate Nepalese students [10] which found no significant difference between age and learning approaches. As the ages of the students were either 19 or 20 years, the study population’s age range is significantly narrower compared to our study. It is unlikely that the relatively small difference in age will have an impact on the learning approaches on the students.

However, another study conducted by Wickramasinghe et al. on a mixture of pre-clinical, clinical and postgraduate students from the University of Colombo, Sri Lanka, concluded that scores for the strategic approach was significantly affected by age among the preclinical students (ρ = 0.206, *p* = 0.002), but no other significant relationship was seen in other groups for any of the approaches [5]. This is in keeping with the results of our study on clinical medical students where age did not significantly impact the learning approaches adopted by students.

Since a significant association between age and learning approach was only noted amongst preclinical students in the other studies and not amongst the students in their clinical years, one explanation for this might be the differences in the format of teaching. During the first two pre-clinical years in medical school, didactic lectures, which promote surface and strategic learning, are the main method of delivery to impart large amounts of medical knowledge, and students’ approaches towards learning are constrained to some degree by the nature of environment and assessment strategy. Whereas in the later years, learning takes place in a clinical environment and more emphasis is placed on problem-based learning (PBL), which is able to increase deep learning [16].

Previous findings reported by Emilia et al. [12] in an Indonesian medical school and Wickramasinghe et al. [5] found that gender was not significantly associated with the predominant learning approach. In the Nepalese study conducted by Shankar et al., there was also no significant difference in scores by gender [10]. While our results were similar, it was interesting to find that the male students in our study are deemed to be less likely than female students to exhibit strategic learning, with *p* value = 0.06 almost reaching statistical significance of 0.05. An important fact to also note is that learning styles may change over a shorter time frame than over the course of a medical degree. Learning styles indeed may change based on the context, environment and topic being learned and is likely a flexible changing trait rather than a fixed innate trait a student possess [23, 24]. Our study is a pilot study and serves as a starting point for student awareness of different learning styles and to start reflecting on adopting more appropriate learning styles in different situations.

Our findings found that age, gender, highest education qualification have no significant association with one’s predominant learning approach. Therefore, further longitudinal studies as well as further studies to monitor and identify key factors that cause students’ learning approach to change through the years would be beneficial for planning and modifying medical curriculum in the medical schools in Singapore. For instance, if factors such as the syllabus structure or learning environment in medical school have been identified to play a crucial role in changing the predominant learning approach of the individual, then efforts can be focused on these areas to encourage students to adopt the deep approach from the start of medical school.

Further studies can also be conducted to investigate the relation between learning approaches and academic performances amongst medical students in Singapore since other studies have suggested a positive correlation between learning approaches and academic performance, where students with deep approach achieved higher performances and vice versa [3]. With the increase in the number of medical students and limited amount of resources and time, finding the solution to encourage independent deep learning amongst the students would be beneficial in the long run, ensuring that students develop the most advantageous learning approach from the start. Hence, motivating medical students towards deep learning would be beneficial in achieving expected long-term goals.

### Limitations

There are limitations to the ASSIST questionnaire despite it being valid and internally consistent. The ASSIST questionnaire is a self-reporting instrument and may not always reflect the true approach to learning of students, especially if they answered the questions in a way that they thought would have been the approved answers [8]. Furthermore, since there were many different questions to check for consistency in the student’s response, this renders it difficult for students to provide certain approved answers, which may ensure that the results obtained are more objective.

We feel that a more critical approach to describing the nature and categorisation of learning approaches could have been taken. The student population may have experienced a range of medical curricula prior to taking part in the study. Out of the three cohorts we included, the students from Singapore Medical schools are likely to have experienced similar curriculums. The third cohort consisting of foreign students on their elective placements might have been exposed to very different medical educational approaches. Though they comprise of only 18.5% of total study population, it would have been worthwhile considering their previous educational experiences and cultural backgrounds.

We attempted to use other tests that allowed for the determination of a single approach such as using paired t-tests. When we tried to use paired t-test for analysis, there was a significant difference between deep (M = 0.40, SD = 0.49) and surface (M = 0.09, SD = 0.28) approaches; t (237) = 7.75, *p* < 0.01) and between strategic (M = 0.51, SD = 0.50) and surface (M = 0.09, SD = 0.28) approaches; t (237) = 9.98, p < 0.01). These results suggest that there is a statistically significant difference between the mean scores of deep versus surface, and strategic versus surface. There was also a borderline significance between deep (M = 0.40, SD = 0.49) and strategic (M = 0.51, SD = 0.50) approaches; t(237) = − 1.70, *p* = 0.09. There was a significant difference between students adopting surface approach and the other two approaches and this was what we expected to identify, since we wanted to determine the percentage of students adopting the surface approach so that we could focus on interventions to reduce this percentage in our future studies.

There are other limitations of this study which include the small sample size and examining a small range of demographic variables. These limitations also do not allow us to make more changes that are specific to the curriculum in order to encourage students to use higher order learning approaches, which are desirable in medical education. However, given these limitations, we felt that there is a need to still conduct and publish this study because it provides initial direction and approach to modifying the curriculum. This is a baseline pilot study with the intention of determining the course of future directions to more specifically tailor interventions in larger scale future studies. We firstly would need to explore whether these learning approaches are related to demographic variables for which we can then more appropriately plan future studies, especially designing interventions and tailoring where best to intervene along the students learning time course.

## Conclusions

This study found that there were no demographic factors that were significantly associated with the predominant learning approaches in a heterogeneous medical student population, and that the majority of students predominantly utilised the deep and strategic learning approach. With the transformation of modern medicine, there are changing demands and expectations of the skills that doctors will need to master, such as deeper thinking and critical analysis during unforeseen and unfamiliar situations or in research. These would require a good understanding of medical knowledge as well as the ability to apply these medical skills and knowledge to different scenarios, which is best brought about by a deep learning approach.

It is thus timely to investigate further how educators can intervene and alter clinical teaching methods to optimize students’ learning based on their preferred learning methods and to encourage a shift towards deep learning.

## Abbreviations

ASI: Approaches to Studying Inventory
ASSIST: Approaches and Study Skills Inventory for Students
CIRB: Centralised Institutional Review Board of SingHealth
Duke-NUS: Duke-NUS Medical School
KKH: KK Women’s and Children’s Hospital
MD: Undergraduate medical program
O&G: Obstetrics and Gynaecology
PBL: Problem-based learning
YLL: National University of Singapore Yong Loo Lin School of Medicine

## References

[1] Entwistle N, Marton F, Hounsell D, Entwistle N (1997). Contrasting perspectives on learning. The experience of learning: implications for teaching and studying in higher education.

[2] Leite WL, Svinicki M, Shi Y (2010). Attempted validation of the scores of the VARK: learning styles inventory with multitrait–multimethod confirmatory factor analysis models. Educ Psychol Meas.

[3] Subasinghe SD, Wanniachchi DN (2009). Approach to learning and the academic performance of a group of medical students–any correlation. Stud Med J.

[4] Trigwell K, Prosser M (1991). Improving the quality of student learning: the influence of learning context and student approaches to learning on learning outcomes. High Educ.

[5] Wickramasinghe DP, Samarasekera DN (2011). Factors influencing the approaches to studying of preclinical and clinical students and postgraduate trainees. BMC Med Educ..

[6] Samarakoon L, Fernando T, Rodrigo C, Rajapakse S (2013). Learning styles and approaches to learning among medical undergraduates and postgraduates. BMC Med Educ.

[7] Shankar PR, Balasubramanium R, Dwivedi NR (2014). Approach to learning of medical students in a Caribbean medical school. Educ Med J.

[8] Reid WA, Evans P, Duvall E (2012). Medical students’ approaches to learning over a full degree programme. Med Educ Online.

[9] Shah DK, Yadav RL, Sharma D, Yadav PK, Sapkota NK, Jha RK, Islam MN (2016). Learning approach among health sciences students in a medical college in Nepal: a cross-sectional study. Adv Med Educ Pract.

[10] Shankar PR, Dubey AK, Binu VS, Subish P, Deshpande VY (2005). Learning styles of preclinical students in a medical college in western Nepal. Kathmandu Univ Med J.

[11] Aaron S, Skakun E (1999). Correlation of students’ characteristics with their learning styles as they begin medical school. Acad Med.

[12] Emilia O, Mulholland H (1991). Approaches to learning of students in an Indonesian medical school. Med Educ.

[13] Davie S (2015). NUS medical school sees more diverse student mix.

[14] Tait H, Entwistle NJ, McCune V, Rust C (1998). ASSIST: a reconceptualisation of the approaches to studying inventory. Improving students as learners.

[15] Gadelrab HF (2011). Factorial structure and predictive validity of approaches and study skills inventory for students (ASSIST) in Egypt: a confirmatory factor analysis. Electron J Res Educ Psychol.

[16] Abedin NF, Jaafar Z, Husain S, Abdullah R (2013). The validity of ASSIST as a measurement of learning approach among MDAB students. Procedia Soc Behav Sci.

[17] Newble DI, Entwistle NJ (1986). Learning styles and approaches: implications for medical education. Med Educ.

[18] Byrne M, Flood B, Willis P (2002). The relationship between learning approaches and learning outcomes: a study of Irish accounting students. Acc Educ.

[19] Cebeci S, Dane S, Kaya M, Yigitoglu R (2013). Medical students’ approaches to learning and study skills. Procedia Soc Behav Sci.

[20] O’Grady G, Choy J. Assessing to foster and measure deep learning in problem based learning. In Holistic Assessment and Problem-based Learning, 5th, Asia-Pacific Conference on PBL. Florida 2008.

[21] Amini M, Tajamul S, Lotfi F, Kariamian Z (2012). A survey on study habits of medical students in shiraz medical school. Future Med Educ J.

[22] Tan T (2014). $1 billion spent on tuition in one year.

[23] Coffield F, Moseley D, Hall E, Ecclestone K. Learning styles and pedagogy in post-16 learning: a systematic and critical review. London: Learning and Skills Research Centre; 2004. http://www.voced.edu.au/content/ngv:13692. Accessed 2 Oct 2017.

[24] Hall E (2016). The tenacity of learning styles: a response to lodge, Hansen, and Cottrell. Learn Res Pract.

